# Clinical value of integrated-signature miRNAs in colorectal cancer: miRNA expression profiling analysis and experimental validation

**DOI:** 10.18632/oncotarget.6065

**Published:** 2015-10-10

**Authors:** XiangJian Chen, KeQing Shi, YuQun Wang, Mei Song, Wu Zhou, HongXiang Tu, Zhuo Lin

**Affiliations:** ^1^ Department of Endoscopic Surgery, The First Affiliated Hospital of Wenzhou Medical University, Wenzhou, Zhejiang, China; ^2^ Department of Infection and Liver Diseases, Liver Research Center, The First Affiliated Hospital of Wenzhou Medical University, Wenzhou, Zhejiang, China; ^3^ Department of Medical Laboratory, The First Affiliated Hospital of Wenzhou Medical University, Wenzhou, Zhejiang, China

**Keywords:** colorectal cancer, microRNA, signature, biomarker, robust rank aggregation

## Abstract

MicroRNA (miRNA) expression profiling of colorectal cancer (CRC) are often inconsistent among different studies. To determine candidate miRNA biomarkers for CRC, we performed an integrative analysis of miRNA expression profiling compared CRC tissues and paired neighboring noncancerous colorectal tissues. Using robust rank aggregation method, we identified a miRNA set of 10 integrated-signature miRNAs. In addition, the qRT-PCR validation demonstrated that 9 miRNAs were consistent dysregulated with the integrative analysis in CRC tissues, 4 miRNAs (miR-21-5p, miR-183-5p, miR-17-5p and miR-20a-5p) were up-regulated expression, and 5 miRNAs (miR-145-5p, miR-195-5p, miR-139-5p, miR-378a-5p and miR-143-3p) were down-regulated expression (all *p* < 0.05). Consistent with the initial analysis, 7 miRNAs were found to be significantly dysregulated in CRC tissues in TCGA data base, 4 miRNAs (miR-21-5p, miR-183-5p, miR-17-5p and miR-20a-5p) were significantly up-regulated expression, and 3 miRNAs (miR-145-5p, miR-139-5p and miR-378a-5p) were significantly down-regulated expression in CRC tissues (all *p* < 0.001). Furthermore, miR-17-5p (*p* = 0.011) and miR-20a-5p (*p* = 0.003) were up-regulated expression in the III/IV tumor stage, miR-145-5p (*p* = 0.028) and miR-195-5p (*p* = 0.001) were significantly increased expression with microscopic vascular invasion in CRC tissues, miR-17-5p (*p* = 0.037) and miR-145-5p (*p* = 0.023) were significantly increased expression with lymphovascular invasion. Moreover, Cox regression analysis of CRC patients in TCGA data base showed miR-20a-5p was correlated with survival (hazard ratio: 1.875, 95%CI: 1.088–3.232, *p* = 0.024). Hence, the finding of current study provides a basic implication of these miRNAs for further clinical application in CRC.

## INTRODUCTION

Colorectal cancer (CRC) is the third most commonly cancer worldwide. The incidence of CRC is 4.4% in developed areas and 1.4% in developing areas [1], and the five year survival rate ranges from 40% to 60% [2]. The survival of the patients relies on early detection significantly, and the prognosis mainly depends on tumor-node-metastasis (TNM) classification [3, 4]. Most of the patients are in poor prognosis while they are often diagnosed at an advanced stage[3]. Although there are various methods to screen biomarkers in CRC, they are often lack of sensitivity or specificity [5-7].

MicroRNAs (miRNAs) belong to a class of small noncoding RNAs that can regulate the expression level of target genes at the post-transcription phase. Growing evidences indicate that miRNAs can serve as ideal biomarkers for cancer detection and accurate predictions of prognosis, as well as targets for treatment [8, 9]. The number of miRNAs has increased largely from 2002 to 2014, and the latest miRBase version (Release 21, 2014) contains 2588 mature human miRNA entries. There are some studies to search biomarkers by analyzing miRNA expression profiling between CRC tissues and paired neighboring noncancerous colorectal tissues, but the results of identification significantly expressed miRNAs are inconsistent or discrepant among each study. There are several reasons to be responsible for the inconsistent results, such as different technological detection platforms, various methods for data processing, constant discovery of new miRNAs and samples from different backgrounds.

To overcome these limitations, we need to integrate their results in an unbiased manner. Robust rank aggregation (RRA) approach has been specifically designed for comparison of several ranked gene lists [10]. The tool looks at how each item is positioned in the ranked lists and compares this to the baseline case where all the preference lists are randomly shuffled. As a result, a *p*-value would be assigned for all items, showing how much better it was positioned in the ranked lists than expected by chance. This *p*-value is used both for reranking the items and deciding their significance. RRA is a suitable and effective solution for identification of statistically significant miRNA integrated-signature and is particularly useful when input experiments are performed by different technological platforms cover different sets of genes and full rankings of miRNAs are not available.

Hence, we executed integrated analysis approach by using RRA method to identify the consistently significant miRNAs in different expression profiling datasets. Moreover, we further validated the consistent up-regulated and down-regulated miRNAs in fresh CRC tissues and TCGA datasets in a clinical setting.

## RESULTS

### Characteristics of the studies and datasets

According to selection criteria, 27 CRC miRNA expression profiling datasets from 24 studies were retrieved from publication. A brief description of the studies is provided in Supplementary Table 1. All studies were reported by the peer-reviewed publications. The studies were published between 2006 and 2014, and 9 studies (more than one third) were published in 2012. Among the 24 studies, 11 studies (PZ [11], RE [12], PP [13], HF [14], GD [15], FK [16], CL [17], CH [18], WA [19], MZ [20], BD [21]) conducted in Europe population, 8 studies (XU [22], LA [23], ZH [24], LX [25], FU [26], CW [27], MY [28], CX [29]) conducted in Asia population and 5 studies (LE [30], SL [31], AR [32], ST [33], CM [34]) conducted in North America population. Distribution of CRC-specific miRNA alterations as reported by primary studies was shown in Supplementary Figure 1.

The number of research samples ranged from 3 to 84 (median 27) across the studies. The average number of miRNAs assayed was 772 (ranging from 156 to 2090) and most of the studies applied various microarray platforms (either commercial or custom). Five studies were focused only on colon cancer and two studies included only rectal cancer. One study (CL) listed the results from different microarray platforms separately and these data were treated as four different input datasets. The pooled datasets included a total of 588 CRC samples and 505 noncancerous tissues samples.

**Figure 1:**
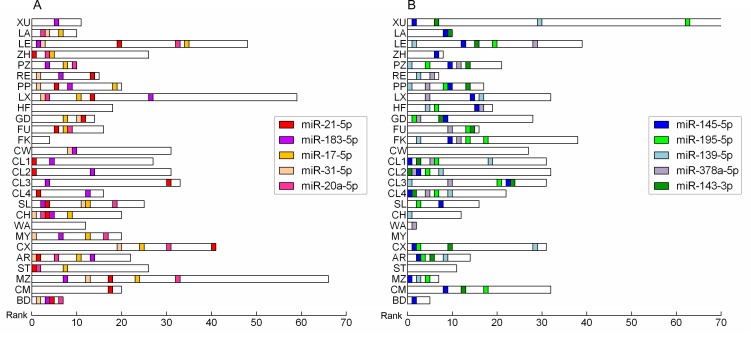
The size of dysregulated miRNA lists in each study Horizontal boxes indicate the number of significantly up-regulated **A.** or down-regulated **B.** miRNAs. The rank scale is shown on the bottom. Positions of CRC integrated-signature miRNAs are highlighted with different colors.

In total, there are 228 significantly up-regulated miRNAs and 230 significantly down-regulated miRNAs reported at least in one study. Additionally, 77 miRNAs were reported as inconsistent alteration. Each study reported with up-regulated miRNAs and down-regulated miRNAs except one study (MY), which reported with only up-regulated miRNAs and no down-regulated ones. The number of significantly dysregulated miRNAs varies across the studies and the top list of miRNAs differs greatly from study to study (Figure 1). Additionally, none of the miRNAs altered consistently across all of the studies.

### Identification integrated-signature miRNAs

A list of statistically significant integrated-signature miRNAs included 5 up-regulated and 5 down-regulated miRNAs were identified in CRC samples compared to noncancerous colorectal tissues using RRA method (Table 1). All of the 10 integrated-signature miRNAs that reached statistical significance after Bonferroni correction were reported by at least 1/2 datasets. The full list of statistically significant miRNAs is provided in Supplementary Table 2. The most significantly up-regulated (miR-21-5p) and down-regulated (miR-145-5p) miRNAs were reported by 19 and 21 datasets, respectively. The direction of expression alteration of integrated-signature miRNAs is consistent across all studies. The majority of the integrated-signature miRNAs belong to the broadly conserved seed families (conserved across most vertebrates).

**Table 1:** Integrated-signature miRNAs in CRC

miRNA	Chromosome location	Corrected *p*-value	Permutation *p*-value	datasets	Seed family	miRNA Cluster
Up-regulation						
miR-21-5p	17q23.2	2.81E-18	1.34E-21	19	miR-21/590-5p	-
miR-183-5p	7q32.2	1.57E-15	7.51E-19	17	miR-183	miR-183/96/182
miR-17-5p	13q31.3	2.44E-10	1.17E-13	15	miR-17/17-5p/20ab/20b-5p/93/106ab/427/518a-3p/519d	miR-17/18a/19a/20a/19b-1/92a-1
miR-31-5p	9p21.3	1.94E-14	9.28E-18	14	miR-31	-
miR-20a-5p	13q31.3	1.66E-08	7.94E-12	14	miR-17/17-5p/20ab/20b-5p/93/106ab/427/518a-3p/519d	miR-17/18a/19a/20a/19b-1/92a-1
Down-regulation						
miR-145-5p	5q32	6.13E-24	2.93E-27	21	miR-145	miR-143/145
miR-195-5p	17p13.1	2.25E-14	1.08E-17	17	miR-15abc/16/16abc/195/322/424/497/1907	miR-195/497
miR-139-5p	17q13.4	5.04E-15	2.41E-18	16	miR-139-5p	-
miR-378a-5p	5q32	2.90E-11	1.39E-14	14	miR-378/422a/378bcdefhi	-
miR-143-3p	5q32	1.29E-10	6.16E-14	14	miR-143/1721/4770	miR-143/145

The 10 integrated-signature miRNA genes are scattered on different chromosome regions and 6 integrated-signature miRNAs belong to the cluster of two or more miRNAs. A cluster is defined as follows: miRNA genes locate at a distance of less than 10 kb and not separated by a transcription unit. MiR-17-5p and miR-20a-5p are all in the chromosomal location of 13q31.3 and belong to the same cluster. MiR-145-5p, miR-378a-5p and miR-143-3p are all in the chromosomal location of 5q32, but miR-378a-5p doesn't belong to the cluster of miR-143/145.

Analysis of studies which consisted of colon or rectum samples only, showed that in colon cancer, there are three up-regulated (miR-21-5p, miR-20a-5p and miR-17-5p) and two down-regulated (miR-145-5p and miR-195-5p) integrated-signature miRNAs reached statistical significance (Bonferroni-corrected *p* = 0.0003, 0.0013, 0.0031, 0.0002 and 0.0254, respectively). In the case of rectal cancer, exception of miR-183-5p and miR-139-5p, the other integrated-signature miRNAs were all reached statistical significance.

### Validation the expression of integrated-signature miRNAs in patients with CRC and clinical significance

**Figure 2:**
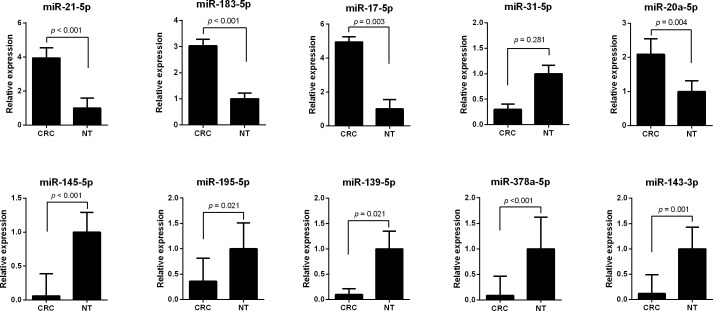
The expression level of integrated-signature miRNAs by qRT-PCR analysis **A.** miR-21-5p, **B.** miR-183-5p, **C.** miR-17-5p, **D.** miR-31-5p, **E.** miR-20a-5p, **F.** miR-145-5p, **G.** miR-195-5p, **H.** miR-139-5p, **I.** miR-378a-5p, **J.** miR-143-3p. CRC: colorectal cancer; NT, noncancerous colorectal tissue.

To validate the expression of the 10 integrated-signature miRNAs that may be the candidate biomarkers for CRC, the expression levels of these miRNAs were compared using qRT-PCR analysis between 11 CRC tissues and the neighboring noncancerous colorectal tissues. The results showed that expression alteration of the miRNAs is consistent with the integrated analysis except miR-31-5p. The expression level of miR-21-5p, miR-183-5p, miR-17-5p and miR-20a-5p were increased more than 2 folds (all *p* < 0.05), whereas miR-145-5p, miR-195-5p, miR-139-5p, miR-378a-5p and miR-143-3p were decreased more than 2 folds in CRC tissues (all *p* < 0.05) (Figure 2). The most up-regulated expressed miRNA was miR-17-5p with a 4.95 fold change (*p* = 0.003) and the most down-regulated expressed miRNA was miR-378a-5p with a 0.09 fold change in CRC tissues compared to noncancerous tissues (*p* < 0.001).

**Figure 3:**
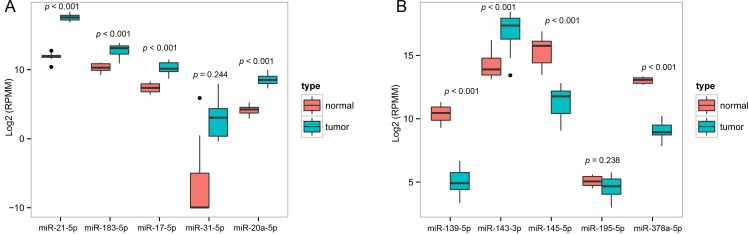
Validation of miRNAs expression in CRC in the TCGA datasets **A.** Upregulated miRNAs expression. **B.** Downregulated miRNAs expression. For boxplots, expression values of miRNAs were log2-transformed and box width was proportional to the square root of sample size in each variant.

Consistent with our initial analysis, 7 miRNAs were found to be significantly dysregulated in CRC tissues in Tumor Cancer Genome Atlas (TCGA) data base, except miR-143-3p, miR-195-5p and miR-31-5p (Figure 3A, Figure 3B). However, the expression changed more than 2-fold was found in miR-21-5p, miR-183-5p, miR-17-5p, miR-20a-5p, miR-145-5p, miR-139-5p and miR-378a-5p (all *p* < 0.001). The TCGA results showed miR-17-5p (*p* = 0.011) and miR-20a-5p (*p* = 0.003) were significantly increased in the III/IV tumor stage comparing to I/II tumor stage (Supplementary Figure 2A, 2B). Patients with lymphovascular invasion had a significantly increased expression of miR-17-5p (*p* = 0.037) and miR-145-5p (*p* = 0.023), while a decreased expression of miR-143-3p (*p* = 0.006) comparing to ones without lymphovascular invasion (Supplementary Figure 3A, 3B). In addition, miR-145-5p (*p* = 0.028) and miR-195-5p (*p* = 0.001) were significantly increased with microscopic vascular invasion (MVI) comparing to non-MVI (Supplementary Figure 4A, 4B).

**Figure 4:**
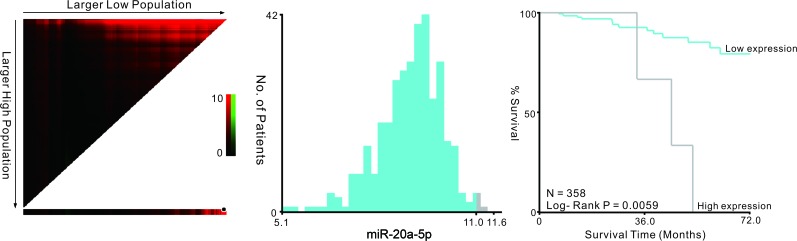
Kaplan-Meier survival analysis by X-tile plots cut-off point The plot showed the chi-squared log-rank values created when the cohort was divided into two groups. The optimal cut-point highlighted by the black circle in the left panels is shown on a histogram of the entire cohort (middle panels) and a Kaplan-Meier plot (right panels).

Furthermore, we used Cox regression analysis of CRC patients in TCGA data base to build a prognostic classifier, by which only miR-20a-5p was selected: miR-20a-5p (hazard ratio [HR]: 1.875, 95%CI: 1.088-3.232, *p* = 0.024). X-tile and K-M survival analysis also showed the miR-20a-5p could predict the clinical outcome of CRC patients in TCGA data base (Figure 4).

### Target prediction and functional analysis

Target genes were obtained from both prediction algorithms and experimentally supported databases. The counts of predicted targets, experimentally validated targets and consensus targets were summarized in Supplementary Figure 5. MiR-17-5p had highest number of consensus targets, whereas miR-378a-5p was the miRNA with smallest number of targets. In addtion, enrichment analyses were performed to elucidate the biological function of consensus target genes. Finally, 139 KEGG pathways, 78 Panther pathways and 978 GO processes were enriched with the miRNAs targets.

**Figure 5:**
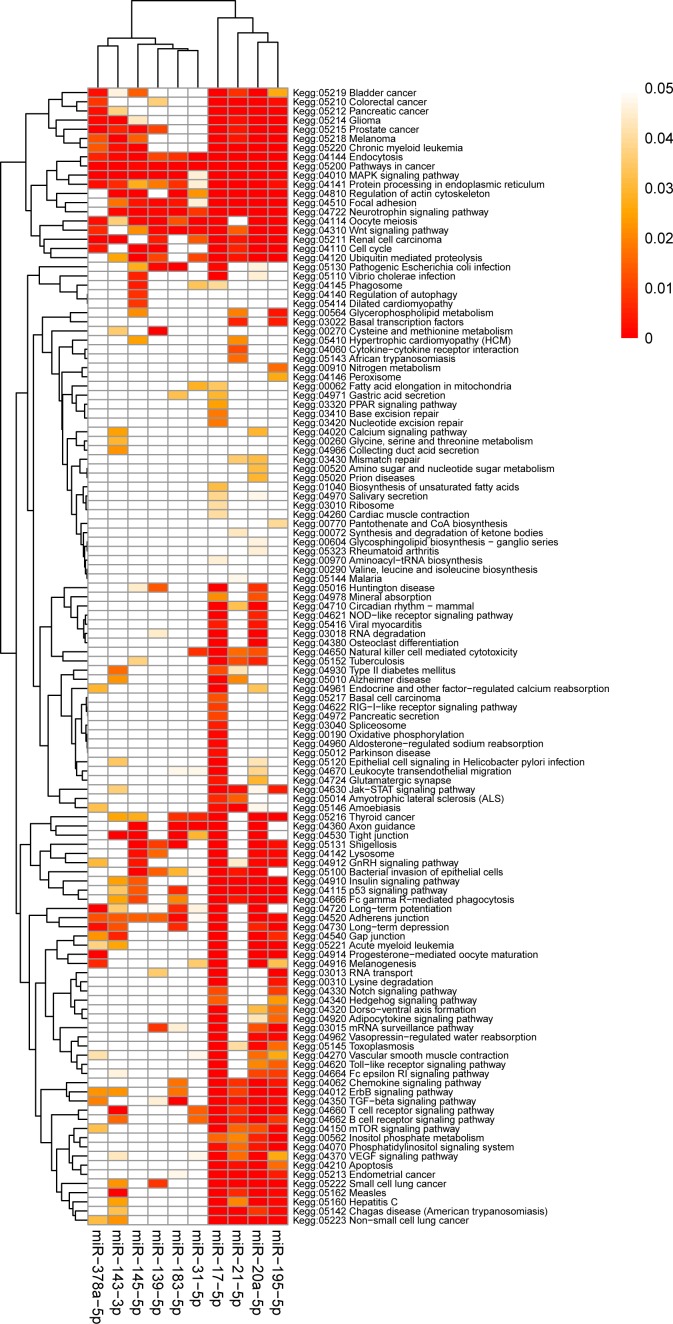
The heatmap of enriched KEGG pathways KEGG pathways with FDR-corrected *p*-value of each integrated-signature miRNA were constructed a heatmap showing the results of pathway enrichment analysis. The intensity of color represents the FDR-corrected *p*-value. Clustering was implemented using Pearson correlation and average linkage method.

Enriched KEGG pathways and Panther pathways were most frequently associated with cell signaling (MAPK signaling pathway, Wnt signaling pathway, EGF receptor signaling pathway, Apoptosis signaling pathway) and tumorigenesis (Pathways in cancer, Angiogenesis, Ras Pathway, p53 pathway), but also with cell mobility and community (regulation of actin cytoskeleton, Focal adhesion) (Table 2). The most enriched GO processes regulated by the miRNAs include regulation of transcription, cell cycle, signal transduction, and apoptotic process. The heatmaps of enriched KEGG pathways and Panther pathways are shown in Figure 5 and Supplementary Figure 6, respectively.

**Table 2:** Ten pathways and GO processes most strongly enriched by targets of integrated -signature miRNAs

Pathways and GO processes	FDR	Targets
KEGG Pathway		
Kegg: 05200 Pathways in cancer	4.61E-42	147
Kegg: 04010 MAPK signaling pathway	5.02E-32	116
Kegg: 04144 Endocytosis	1.80E-28	92
Kegg: 04722 Neurotrophin signaling pathway	1.38E-26	69
Kegg: 04120 Ubiquitin mediated proteolysis	2.72E-26	72
Kegg: 04810 Regulation of actin cytoskeleton	1.63E-25	92
Kegg: 04510 Focal adhesion	6.65E-23	85
Kegg: 04310 Wnt signaling pathway	2.72E-22	71
Kegg: 05215 Prostate cancer	5.57E-22	52
Kegg: 04141 Protein processing in endoplasmic reticulum	8.64E-22	73
Panther Pathway		
Panther: P00034 Integrin signalling pathway	2.73E-20	71
Panther: P00005 Angiogenesis	4.65E-20	70
Panther: P00047 PDGF signaling pathway	6.65E-20	62
Panther: P00018 EGF receptor signaling pathway	7.65E-20	58
Panther: P00021 FGF signaling pathway	8.20E-19	55
Panther: P04393 Ras Pathway	1.36E-16	40
Panther: P00057 Wnt signaling pathway	1.53E-16	94
Panther: P00046 Oxidative stress response	3.45E-12	29
Panther: P00059 p53 pathway	4.14E-11	36
Panther: P00006 Apoptosis signaling pathway	9.78E-11	43
GO processes		
GO: 0006355 regulation of transcription, DNA-dependent (BP)	4.03E-113	572
GO: 0045892 negative regulation of transcription, DNA-dependent (BP)	2.26E-51	182
GO: 0045893 positive regulation of transcription, DNA-dependent (BP)	4.82E-45	190
GO: 0045944 positive regulation of transcription from RNA polymerase II promoter (BP)	5.96E-45	217
GO: 0007049 cell cycle (BP)	9.89E-45	181
GO: 0007165 signal transduction (BP)	1.25E-43	344
GO: 0006915 apoptotic process (BP)	4.42E-43	217
GO: 0000122 negative regulation of transcription from RNA polymerase II promoter (BP)	1.70E-41	171
GO: 0044419 interspecies interaction between organisms (BP)	3.75E-40	146
GO: 0015031 protein transport (BP)	1.56E-37	164

**Figure 6:**
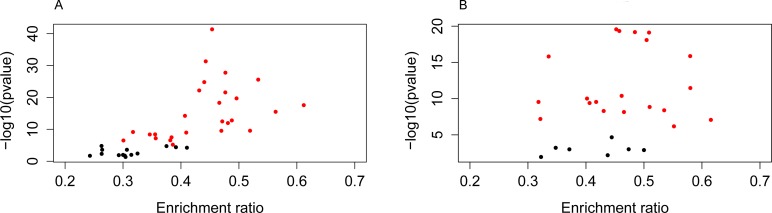
The most significantly enriched pathways related with CRC The red dots indicate KEGG pathways **A.** and Panther pathways **B.** highly related with CRC (enrichment ratio > 0.3, *p* < 0.00001).

Furthermore, to evaluate association between these pathways and CRC, the publications which described CRC related constituent objects in the pathways were searched in PubMed. The pathways in which the constituent objects supported were considered to be CRC-related. After text mining, 42 KEGG pathways and 28 Panther pathways were found to be CRC-related. To visualize the most significantly enriched pathways, volcano plots were constructed by plotting the -log10 of *p*-value *versus* gene enrichment ratio. Finally, 26 of the 42 KEGG pathways and 21 of the 28 Panther pathways were highly related with CRC (enrichment ratio > 0.3, *p*-value < 0.00001) (Supplementary Table 3, Figure 6).

## DISCUSSION

The most common drawback of miRNA expression profiling studies is inconsistency among different studies. A reasonable solution to the problem is to determine the miRNAs which were consistently reported among the different miRNA expression profiling studies and expressed in a consistent orientation. Systematic review or meta-analysis has been done previously to determine differentially expressed genes in cancer at the gene level [35, 36]. However, such rigorous approach is often not possible due to the lack of cross-platform standardization of miRNA profiling technologies or the unavailability of raw data. In this study, we analyzed CRC-specific miRNAs derived from independent expression profiling experiments using RRA method. The miRNAs would be re-ranked and their significance would be re-decided. We identified an integrated-signature of 5 up-regulated and 5 down-regulated miRNAs from 27 expression profiling datasets included more than 1000 CRC tissue and noncancerous tissue samples.

To determine whether the 10 integrated-signature miRNAs have clinical values as biomarkers in CRC, we also performed a validation experiment. Our data confirmed the expression changes of 9 miRNAs were consistent with our integrated analysis. These results of our validation experiment have further supported the findings obtained in the present integrated analysis. Consistent with our initial analysis, 7 miRNAs (miR-21-5p, miR-183-5p, miR-17-5p, miR-20a-5p, miR-145-5p, miR-139-5p and miR-378a-5p) were found to be significantly dysregulated in TCGA data base. In this study, as was validated by qRT-PCR and TCGA datasets, the expression changes of the 7 miRNAs were all consistent with integrated analysis. Therefore, this miRNA panel might be novel potential biomarkers for the diagnosis of CRC.

The integrated-signature miRNAs are key regulatory factors of the oncogenic process. This is supported by KEGG and Panther pathways enrichment analyses of targets of miRNAs, which indicate very strong impact on pathways such as: Pathways in cancer, MAPK signaling pathway, Wnt signaling pathway, EGF receptor signaling pathway, Apoptosis signaling pathway (Table 2). In addition, the association between these pathways and CRC are highly related by publication analysis. Therefore, these miRNAs may be good candidate therapeutic targets in CRC. Further studies could be performed to evaluate the clinical values of the miRNA expression signature in CRC.

The most consistently reported miRNA of up-regulation in our integrated analysis is miR-21-5p, which is an oncogenic factor and often alters expression in cancers. It is shown that miR-21-5p directly target the tumor-suppressor PTEN, chemokine gene CCL20 and PDCD4 [37-39]. High level of miR-21-5p is associated with worse survival and poor response to chemotherapy in CRC patients [40]. Furthermore, miRNA-21-5p can induce resistance to 5-fluorouracil by down-regulating human DNA MutS homolog 2 (hMSH2), which was a core mismatch repair recognition protein complex [41]. Additionally, miR-21-5p expression level showed significant association with depth of invasion, lymphatic and venous invasion, liver metastasis and Dukes’ stage [42]. The most consistently reported down-regulated miRNA is miR-145-5p which was shown to possess anti-tumorigenic activity, including involvement in various cancer-related processes such as proliferation, invasion and migration. It is showed that miR-145-5p directly targeted catenin δ-1, contributing to the aberrant translocation of β-catenin in the Wnt/β-catenin signaling pathway to suppress human colon cancer cells [43]. Moreover, several genes have been shown to be direct target by miR-145-5p in CRC [44-48], and majority of these genes are oncosuppression. Additionally, patients with a low miR-145-5p expression level had significantly more often a worse response to neoadjuvant chemoradiotherapy compared to patients with a high expression level of miR-145-5p [49].

It is showed that miR-183-5p, miR-17-5p and miR-20a-5p are all oncogenic miRNAs that regulates tumorigenesis and progression. MiR-17-5p and miR-20a-5p are located in a cluster in the 13q31.3 chromosomal region, which combine with 8q24 could convert colorectal adenoma to adenocarcinoma [50]. Previous publications have demonstrated that up-regulated miR-17-5p and miR-20a-5p could promoted cell proliferation, tumour growth and cell cycle progression by targeting the RND3 and BIM tumour suppressor gene, respectively [51, 52]. Additionally, miR-183-5p functions as an oncogene by targeting the transcription factor EGR1 and promoting colon cancer cells migration [53]. MiR-139-5p and miR-378a-5p are all anti-tumor miRNAs that exert tumor suppressor function in CRC. MiR-139-5p promotes cell cycle arrest in G0/G1 phase by targeting NOTCH1 in colorectal cancer [54], and miR-378a-5p inhibits cell growth and the G1/S transition by targeting CDC40 in colorectal cancer [55].

Although our analysis was limited to comparison and validation between tumor and noncancerous tissue only, the significantly and consistently reported miRNAs could be used as potential diagnostic biomarkers. In a clinical setting, sufficient sensitivity and specificity of the panel of miRNAs should be determined in the further well designed clinical studies. Furthermore, targets prediction and functional enrichment analysis may provide a clue for elucidating the role of miRNAs in tumorigenesis of CRC and the precise underlying mechanisms. Taken together, the findings of the current study may have substantial clinical significance or implications.

In conclusion, a CRC associated miRNA expression signature, consisting of 7 highly significant and consistently dysregulated miRNAs, were identified in our integrated analysis and validation study, which may be potential candidate biomarkers for CRC. The rigorous evaluation of integrated-miRNA signature and functional enrichment analysis of their targets were promising them as candidates for diagnostic biomarkers of CRC. Further clinical and mechanistic studies focusing on these miRNAs are required for their clinical significance and the underlying mechanisms in tumorigenesis of CRC.

## MATERIALS AND METHODS

### Studies selection and analysis

CRC miRNA expression profiling studies were identified through Web of Science (www.isiknowledge.com) and PubMed (www.ncbi.nlm.nih.gov/pubmed) using search term: ((mirna OR microrna or mirnas or micrornas) AND (profile or profiles or profiling or pattern) AND (colorectal OR colon OR rectal) AND (cancer OR tumor OR adenocarcinoma OR neoplasia)). The last search was performed in December 15 2014. To ensure that relevant studies were not missed, search was also performed in Gene Expression Omnibus (GEO, www.ncbi.nlm.nih.gov/gds) and ArrayExpress (www.ebi.ac.uk/arrayexpress).

Full texts of studies published in English language were involved in our analysis, and only original experimental studies that screened different miRNAs between CRC and paired adjacent noncancerous tissue in human were included for further evaluation. Studies analyzing the individual preselected candidate miRNAs or using only cell lines as the research samples were excluded. Studies that profiled different expression miRNAs only in histological subtypes but did not include noncancerous tissue were also excluded.

The lists of miRNAs that had statistically significant changes in expression level were accurate extracted from each eligible study. In case the miRNA lists was not available from the publications, the authors were contacted directly. All miRNA names were standardized according to miRBase version 21, and viral miRNAs and non-miRNA probes were excluded from integrated analysis.

To identify miRNAs that are ranked consistently better than expected by chance, we used RRA method implemented as a RobustRankAggreg package in R software (version 3.1.3). Analysis was repeated 10,000 times to assess the stability of acquired *p*-values by one random miRNA list removed from the analysis each time. Acquired *p*-values from each round were finally averaged for each miRNA.

### Validation of the integrated-signature miRNAs

To validate the expression of integrated-signature miRNAs, fresh CRC tissues and paired neighboring noncancerous colorectal tissues were obtained from 11 patients by experienced surgeons and examined by experienced pathologists at the First Affiliated Hospital of Wenzhou Medical University (the detailed information of patients is provided in Table 3). Informed consents were obtained from all the patients and the study was approved by the Institutional Review Board. The samples were immediately frozen in liquid nitrogen in 10 minutes after surgical resection and were then stored at −80°C temperature until RNA extraction.

**Table 3:** Clinical features of 11 CRC patients for validation test

Case No.	Age	Gender	Location	Differentiation	Tumor morphology	Lymph node	Metastasis	TNM Stage
CRC #1	45	Male	Rectum	Moderate	Tis	N_0_	M_0_	0
CRC #2	65	Male	Colon	Moderate	T2	N_0_	M_0_	I
CRC #3	67	Male	Colon	Moderate	T3	N_2b_	M_0_	IIIC
CRC #4	75	Female	Colon	Moderate	T3	N_0_	M_0_	IIA
CRC #5	62	Male	Rectum	Moderate	T3	N_2a_	M_1a_	IVA
CRC #6	58	Male	Rectum	Moderate	T3	N_1b_	M_0_	IIIB
CRC #7	32	Male	Rectum	Moderate	T2	N_0_	M_0_	I
CRC #8	69	Male	Rectum	Moderate	T3	N_2b_	M_0_	IIIC
CRC #9	58	Female	Rectum	Moderate	T3	N_1a_	M_0_	IIIB
CRC #10	59	Male	Colon	High	T2	N_2a_	M_1a_	IVA
CRC #11	66	Male	Colon	Moderate	T2	N_0_	M_0_	I

Total RNA was extracted and isolated using miRNeasy Micro Kit (QIAGEN) following the manufacturer's instructions. SYBR-Green qRT-PCR assay was used for miRNA quantification. In brief, 40 ng of total RNA containing miRNA was polyadenylated by poly (A) polymerase and then reverse transcribed to cDNA using miScript Reverse Transcription kit (QIAGEN) according to the manufacturer's instructions. Real-time qPCR was performed using miScript SYBR-Green PCR kit (QIAGEN) containing universal primer in ABI 7500 PCR system (Applied Biosystems). The primers of each dysregulated miRNAs were listed in Supplementary Table 4. Each reaction volume contains 4 μl of cDNA, 0.5 mM of each primer and SYBR-Green PCR Master mix in a final 20 μl volume. The amplification program was: 95°C for 2 min for one cycle, then 94°C for 20 sec with 60°C for 35 sec for 45 cycles, and melting curve analyses were performed at the end of the cycles. The expression levels of miRNAs were normalized to RNU6B. The relative expression of each miRNA was calculated using the 2^−ΔΔCt^ method. Student's *t*-test was used to compare miRNA expression values of CRC and noncancerous colorectal tissues, and *p* < 0.05 indicated significant difference.

The results of qRT-PCR were validated in the TCGA datasets. The miRNA expression data and corresponding clinical information for CRC datasets were downloaded from TCGA data portal in June 2015(Supplementary Table 5). TCGA data are classified by data type (clinical, mutations, gene expression) and data level, to allow structured access to this resource with appropriate patient privacy protection. This study meets the publication guidelines provided by TCGA. The miRNA expression profiling was performed using the Illumina HiSeq 2000 miRNA sequencing platforms (Illumina Inc, San Diego, CA). The miRNA expression level was demonstrated as reads per million miRNA mapped data. The miRNA expression analyses were performed using BRB-ArrayTools (version 4.4). In brief, the miRNAs with missing data exceeded 10% of all subjects were excluded from the datasets and the expression level of each individual miRNA was log2-transformed for further analysis.

The prognosis of CRC strongly depends upon tumor stage, lymphovascular invasion and microscopic vascular invasion (MVI). Therefore, the differences of miRNAs expression level were also tested with or without lymphovascular invasion, presence of MVI or not, and different tumor stages in the TCGA datasets. The statistical analyses were performed using the SPSS 18.0 (SPSS Inc.). Statistical significance was defined as *p* < 0.05. For survival analysis, we used the Kaplan-Meier method to analysis the correlation between overall survival and the miRNAs, and the log-rank test was used to compare survival curves. The optimum cut-off value for the miRNAs using X-tile plots based on the association with mortality of the patients. X-tile plots provide a single and intuitive method to assess the association between variables and survival. The X-tile program can automatically select the optimum data cut point according to the highest χ2 value (minimum *p*-value) defined by Kaplan-Meier survival analysis and log-rank test [56]. We did the X-tile plots using the X-tile software version 3.6.1 (Yale University School of Medicine, New Haven, CT, USA).

### Target prediction

The potential targets of integrated-signature miRNAs were predicted by five different target prediction algorithms: TargetScan v6.2 (http://www.targetscan.org/vert_61/), DIANA-microT-CDS v5.0 (http://diana.imis.athena-innovation.gr/DianaTools/index.php?r=microT_CDS/index), miRanda (http://www.microrna.org/microrna/getMirna-Form.do), miRDB (http://mirdb.org/miRDB/) and PicTar (http://pictar.mdc-berlin.de/cgi-bin/PicTar_vertebrate.cgi). Only genes with target sites in 3′ UTR were selected. Validated target genes were obtained from starBase v2.0 database (http://starbase.sysu.edu.cn/mirMrna.php) based on CLIP-Seq and TarBase v7.0 database (http://diana.imis.athena-innovation.gr/DianaTools/index.php?r=tarbase/index). Consensus targets were defined as genes predicted by at least three algorithms plus validated targets.

### Enrichment analysis

Enrichment analyses for KEGG, Panther pathways and GO processes were performed with GeneCodis web tool (http://genecodis.cnb.csic.es/). Consensus target genes for each integrated-signature miRNA were used as input data and then acquired false discovery rate (FDR)-corrected *p*-values of each pathway. Clustering of the heatmap was acquired by applying a pheatmap package in R software (version 3.1.3) based on Pearson correlation and average linkage. Furthermore, the association between the pathways affected by altered expression of miRNAs and CRC was evaluated.

## SUPPLEMENTARY MATERIAL FIGURES AND TABLES



## Abbreviations

CRC: colorectal cancer
FDR: false discovery rate
HR: hazard ratio
miRNAs: microRNAs
MVI: microscopic vascular invasion
RRA: robust rank aggregation
TCGA: Tumor Cancer Genome Atlas
TNM: tumor-node-metastasis.

## References

[1] Jemal A, Bray F, Center MM, Ferlay J, Ward E, Forman D (2011). Global cancer statistics. CA: a cancer journal for clinicians.

[2] Weitz J, Koch M, Debus J, Hohler T, Galle PR, Buchler MW (2005). Colorectal cancer. Lancet.

[3] McPhail S, Johnson S, Greenberg D, Peake M, Rous B (2015). Stage at diagnosis and early mortality from cancer in England. Br J Cancer.

[4] Hu H, Krasinskas A, Willis J (2011). Perspectives on current tumor-node-metastasis (TNM) staging of cancers of the colon and rectum. Seminars in oncology.

[5] Fung KY, Tabor B, Buckley MJ, Priebe IK, Purins L, Pompeia C, Brierley GV, Lockett T, Gibbs P, Tie J, McMurrick P, Moore J, Ruszkiewicz A, Nice E, Adams TE, Burgess A (2015). Blood-based protein biomarker panel for the detection of colorectal cancer. PLoS One.

[6] Zhang A, Sun H, Yan G, Wang P, Han Y, Wang X (2014). Metabolomics in diagnosis and biomarker discovery of colorectal cancer. Cancer letters.

[7] Barderas R, Babel I, Casal JI (2010). Colorectal cancer proteomics, molecular characterization and biomarker discovery. Proteomics Clinical applications.

[8] Berindan-Neagoe I, Monroig Pdel C, Pasculli B, Calin GA (2014). MicroRNAome genome: a treasure for cancer diagnosis and therapy. CA: a cancer journal for clinicians.

[9] Phuah NH, Nagoor NH (2014). Regulation of microRNAs by natural agents: new strategies in cancer therapies.

[10] Vosa U, Kolde R, Vilo J, Metspalu A, Annilo T (2014). Comprehensive meta-analysis of microRNA expression using a robust rank aggregation approach. Methods in molecular biology (Clifton, NJ).

[11] Pizzini S, Bisognin A, Mandruzzato S, Biasiolo M, Facciolli A, Perilli L, Rossi E, Esposito G, Rugge M, Pilati P, Mocellin S, Nitti D, Bortoluzzi S, Zanovello P (2013). Impact of microRNAs on regulatory networks and pathways in human colorectal carcinogenesis and development of metastasis. BMC genomics.

[12] Reid JF, Sokolova V, Zoni E, Lampis A, Pizzamiglio S, Bertan C, Zanutto S, Perrone F, Camerini T, Gallino G, Verderio P, Leo E, Pilotti S, Gariboldi M, Pierotti MA (2012). miRNA profiling in colorectal cancer highlights miR-1 involvement in MET-dependent proliferation. Molecular cancer research: MCR.

[13] Piepoli A, Tavano F, Copetti M, Mazza T, Palumbo O, Panza A, di Mola FF, Pazienza V, Mazzoccoli G, Biscaglia G, Gentile A, Mastrodonato N, Carella M, Pellegrini F, di Sebastiano P, Andriulli A (2012). Mirna expression profiles identify drivers in colorectal and pancreatic cancers. PLoS One.

[14] Hamfjord J, Stangeland AM, Hughes T, Skrede ML, Tveit KM, Ikdahl T, Kure EH (2012). Differential expression of miRNAs in colorectal cancer: comparison of paired tumor tissue and adjacent normal mucosa using high-throughput sequencing. PLoS One.

[15] Gaedcke J, Grade M, Camps J, Sokilde R, Kaczkowski B, Schetter AJ, Difilippantonio MJ, Harris CC, Ghadimi BM, Moller S, Beissbarth T, Ried T, Litman T (2012). The rectal cancer microRNAome—microRNA expression in rectal cancer and matched normal mucosa. Clinical cancer research: an official journal of the American Association for Cancer Research.

[16] Faltejskova P, Svoboda M, Srutova K, Mlcochova J, Besse A, Nekvindova J, Radova L, Fabian P, Slaba K, Kiss I, Vyzula R, Slaby O (2012). Identification and functional screening of microRNAs highly deregulated in colorectal cancer. Journal of cellular and molecular medicine.

[17] Callari M, Dugo M, Musella V, Marchesi E, Chiorino G, Grand MM, Pierotti MA, Daidone MG, Canevari S, De Cecco L (2012). Comparison of microarray platforms for measuring differential microRNA expression in paired normal/cancer colon tissues. PLoS One.

[18] Chang KH, Miller N, Kheirelseid EA, Lemetre C, Ball GR, Smith MJ, Regan M, McAnena OJ, Kerin MJ (2011). MicroRNA signature analysis in colorectal cancer: identification of expression profiles in stage II tumors associated with aggressive disease. International journal of colorectal disease.

[19] Wang YX, Zhang XY, Zhang BF, Yang CQ, Chen XM, Gao HJ (2010). Initial study of microRNA expression profiles of colonic cancer without lymph node metastasis. Journal of digestive diseases.

[20] Monzo M, Navarro A, Bandres E, Artells R, Moreno I, Gel B, Ibeas R, Moreno J, Martinez F, Diaz T, Martinez A, Balague O, Garcia-Foncillas J (2008). Overlapping expression of microRNAs in human embryonic colon and colorectal cancer. Cell research.

[21] Bandres E, Cubedo E, Agirre X, Malumbres R, Zarate R, Ramirez N, Abajo A, Navarro A, Moreno I, Monzo M, Garcia-Foncillas J (2006). Identification by Real-time PCR of 13 mature microRNAs differentially expressed in colorectal cancer and non-tumoral tissues. Molecular cancer.

[22] Xu XH, Wu XB, Wu SB, Liu HB, Chen R, Li Y (2014). Identification of miRNAs differentially expressed in clinical stages of human colorectal carcinoma-an investigation in Guangzhou, China. PLoS One.

[23] Liang G, Li J, Sun B, Li S, Lu L, Wang Y, Chen B, Xiao Z (2014). Deep sequencing reveals complex mechanisms of microRNA deregulation in colorectal cancer. International journal of oncology.

[24] Zhang JX, Song W, Chen ZH, Wei JH, Liao YJ, Lei J, Hu M, Chen GZ, Liao B, Lu J, Zhao HW, Chen W, He YL, Wang HY, Xie D, Luo JH (2013). Prognostic and predictive value of a microRNA signature in stage II colon cancer: a microRNA expression analysis. The Lancet Oncology.

[25] Li X, Zhang G, Luo F, Ruan J, Huang D, Feng D, Xiao D, Zeng Z, Chen X, Wu W (2012). Identification of aberrantly expressed miRNAs in rectal cancer. Oncology reports.

[26] Fu J, Tang W, Du P, Wang G, Chen W, Li J, Zhu Y, Gao J, Cui L (2012). Identifying microRNA-mRNA regulatory network in colorectal cancer by a combination of expression profile and bioinformatics analysis. BMC systems biology.

[27] Chen WC, Lin MS, Ye YL, Gao HJ, Song ZY, Shen XY (2012). microRNA expression pattern and its alteration following celecoxib intervention in human colorectal cancer. Experimental and therapeutic medicine.

[28] Motoyama K, Inoue H, Takatsuno Y, Tanaka F, Mimori K, Uetake H, Sugihara K, Mori M (2009). Over- and under-expressed microRNAs in human colorectal cancer. International journal of oncology.

[29] Chen X, Guo X, Zhang H, Xiang Y, Chen J, Yin Y, Cai X, Wang K, Wang G, Ba Y, Zhu L, Wang J, Yang R, Zhang Y, Ren Z, Zen K (2009). Role of miR-143 targeting KRAS in colorectal tumorigenesis. Oncogene.

[30] Li E, Ji P, Ouyang N, Zhang Y, Wang XY, Rubin DC, Davidson NO, Bergamaschi R, Shroyer KR, Burke S, Zhu W, Williams JL (2014). Differential expression of miRNAs in colon cancer between African and Caucasian Americans: implications for cancer racial health disparities. International journal of oncology.

[31] Slattery ML, Wolff E, Hoffman MD, Pellatt DF, Milash B, Wolff RK (2011). MicroRNAs and colon and rectal cancer: differential expression by tumor location and subtype. Genes, chromosomes & cancer.

[32] Arndt GM, Dossey L, Cullen LM, Lai A, Druker R, Eisbacher M, Zhang C, Tran N, Fan H, Retzlaff K, Bittner A, Raponi M (2009). Characterization of global microRNA expression reveals oncogenic potential of miR-145 in metastatic colorectal cancer. BMC cancer.

[33] Schetter AJ, Leung SY, Sohn JJ, Zanetti KA, Bowman ED, Yanaihara N, Yuen ST, Chan TL, Kwong DL, Au GK, Liu CG, Calin GA, Croce CM, Harris CC (2008). MicroRNA expression profiles associated with prognosis and therapeutic outcome in colon adenocarcinoma. Jama.

[34] Cummins JM, He Y, Leary RJ, Pagliarini R, Diaz LA, Sjoblom T, Barad O, Bentwich Z, Szafranska AE, Labourier E, Raymond CK, Roberts BS, Juhl H, Kinzler KW, Vogelstein B, Velculescu VE (2006). The colorectal microRNAome. Proc Natl Acad Sci U S A.

[35] Griffith OL, Melck A, Jones SJ, Wiseman SM (2006). Meta-analysis and meta-review of thyroid cancer gene expression profiling studies identifies important diagnostic biomarkers. Journal of clinical oncology: official journal of the American Society of Clinical Oncology.

[36] Chan SK, Griffith OL, Tai IT, Jones SJ (2008). Meta-analysis of colorectal cancer gene expression profiling studies identifies consistently reported candidate biomarkers. Cancer epidemiology, biomarkers & prevention: a publication of the American Association for Cancer Research, cosponsored by the American Society of Preventive Oncology.

[37] Xiong B, Cheng Y, Ma L, Zhang C (2013). MiR-21 regulates biological behavior through the PTEN/PI-3 K/Akt signaling pathway in human colorectal cancer cells. International journal of oncology.

[38] Peacock O, Lee AC, Cameron F, Tarbox R, Vafadar-Isfahani N, Tufarelli C, Lund JN (2014). Inflammation and MiR-21 pathways functionally interact to downregulate PDCD4 in colorectal cancer. PLoS One.

[39] Vicinus B, Rubie C, Stegmaier N, Frick VO, Kolsch K, Kauffels A, Ghadjar P, Wagner M, Glanemann M (2013). miR-21 and its target gene CCL20 are both highly overexpressed in the microenvironment of colorectal tumors: significance of their regulation. Oncology reports.

[40] Oue N, Anami K, Schetter AJ, Moehler M, Okayama H, Khan MA, Bowman ED, Mueller A, Schad A, Shimomura M, Hinoi T, Aoyagi K, Sasaki H, Okajima M, Ohdan H, Galle PR (2014). High miR-21 expression from FFPE tissues is associated with poor survival and response to adjuvant chemotherapy in colon cancer. International journal of cancer Journal international du cancer.

[41] Valeri N, Gasparini P, Braconi C, Paone A, Lovat F, Fabbri M, Sumani KM, Alder H, Amadori D, Patel T, Nuovo GJ, Fishel R, Croce CM (2010). MicroRNA-21 induces resistance to 5-fluorouracil by down-regulating human DNA MutS homolog 2 (hMSH2). Proc Natl Acad Sci U S A.

[42] Fukushima Y, Iinuma H, Tsukamoto M, Matsuda K, Hashiguchi Y (2015). Clinical significance of microRNA-21 as a biomarker in each Dukes’ stage of colorectal cancer. Oncology reports.

[43] Yamada N, Noguchi S, Mori T, Naoe T, Maruo K, Akao Y (2013). Tumor-suppressive microRNA-145 targets catenin delta-1 to regulate Wnt/beta-catenin signaling in human colon cancer cells. Cancer letters.

[44] Su J, Liang H, Yao W, Wang N, Zhang S, Yan X, Feng H, Pang W, Wang Y, Wang X, Fu Z, Liu Y, Zhao C, Zhang J, Zhang CY, Zen K (2014). MiR-143 and MiR-145 regulate IGF1R to suppress cell proliferation in colorectal cancer. PLoS One.

[45] Feng Y, Zhu J, Ou C, Deng Z, Chen M, Huang W, Li L (2014). MicroRNA-145 inhibits tumour growth and metastasis in colorectal cancer by targeting fascin-1. Br J Cancer.

[46] Yin Y, Yan ZP, Lu NN, Xu Q, He J, Qian X, Yu J, Guan X, Jiang BH, Liu LZ (2013). Downregulation of miR-145 associated with cancer progression and VEGF transcriptional activation by targeting N-RAS and IRS1. Biochimica et biophysica acta.

[47] Xu Q, Liu LZ, Qian X, Chen Q, Jiang Y, Li D, Lai L, Jiang BH (2012). MiR-145 directly targets p70S6K1 in cancer cells to inhibit tumor growth and angiogenesis. Nucleic acids research.

[48] Pagliuca A, Valvo C, Fabrizi E, di Martino S, Biffoni M, Runci D, Forte S, De Maria R, Ricci-Vitiani L (2013). Analysis of the combined action of miR-143 and miR-145 on oncogenic pathways in colorectal cancer cells reveals a coordinate program of gene repression. Oncogene.

[49] Drebber U, Lay M, Wedemeyer I, Vallbohmer D, Bollschweiler E, Brabender J, Monig SP, Holscher AH, Dienes HP, Odenthal M (2011). Altered levels of the onco-microRNA 21 and the tumor-supressor microRNAs 143 and 145 in advanced rectal cancer indicate successful neoadjuvant chemoradiotherapy. International journal of oncology.

[50] Diosdado B, van de Wiel MA, Terhaar Sive Droste JS, Mongera S, Postma C, Meijerink WJ, Carvalho B, Meijer GA (2009). MiR-17-92 cluster is associated with 13q gain and c-myc expression during colorectal adenoma to adenocarcinoma progression. Br J Cancer.

[51] Luo H, Zou J, Dong Z, Zeng Q, Wu D, Liu L (2012). Up-regulated miR-17 promotes cell proliferation, tumour growth and cell cycle progression by targeting the RND3 tumour suppressor gene in colorectal carcinoma. The Biochemical journal.

[52] Tsuchida A, Ohno S, Wu W, Borjigin N, Fujita K, Aoki T, Ueda S, Takanashi M, Kuroda M (2011). miR-92 is a key oncogenic component of the miR-17-92 cluster in colon cancer. Cancer science.

[53] Cottonham CL, Kaneko S, Xu L (2010). miR-21 and miR-31 converge on TIAM1 to regulate migration and invasion of colon carcinoma cells. The Journal of biological chemistry.

[54] Zhang L, Dong Y, Zhu N, Tsoi H, Zhao Z, Wu CW, Wang K, Zheng S, Ng SS, Chan FK, Sung JJ, Yu J (2014). microRNA-139-5p exerts tumor suppressor function by targeting NOTCH1 in colorectal cancer. Molecular cancer.

[55] Wang KY, Ma J, Zhang FX, Yu MJ, Xue JS, Zhao JS (2014). MicroRNA-378 inhibits cell growth and enhances L-OHP-induced apoptosis in human colorectal cancer. IUBMB life.

[56] Camp RL, Dolled-Filhart M, Rimm DL (2004). X-tile: a new bio-informatics tool for biomarker assessment and outcome-based cut-point optimization. Clinical cancer research: an official journal of the American Association for Cancer Research.

