# Supplementary material for: Clinical value of integrated-signature miRNAs in colorectal cancer: miRNA expression profiling analysis and experimental validation
Source: Oncotarget. 2015 Oct 10;6(35):37544–56. doi: 10.18632/oncotarget.6065 (PMC4741947; doi:10.18632/oncotarget.6065)
Supplement: Supplementary file 1 [file oncotarget-06-37544-s001.pdf]

Clinical value of integrated-signature miRNAs in colorectal cancer: miRNA expression profiling analysis and experimental validation

Supplementary Material

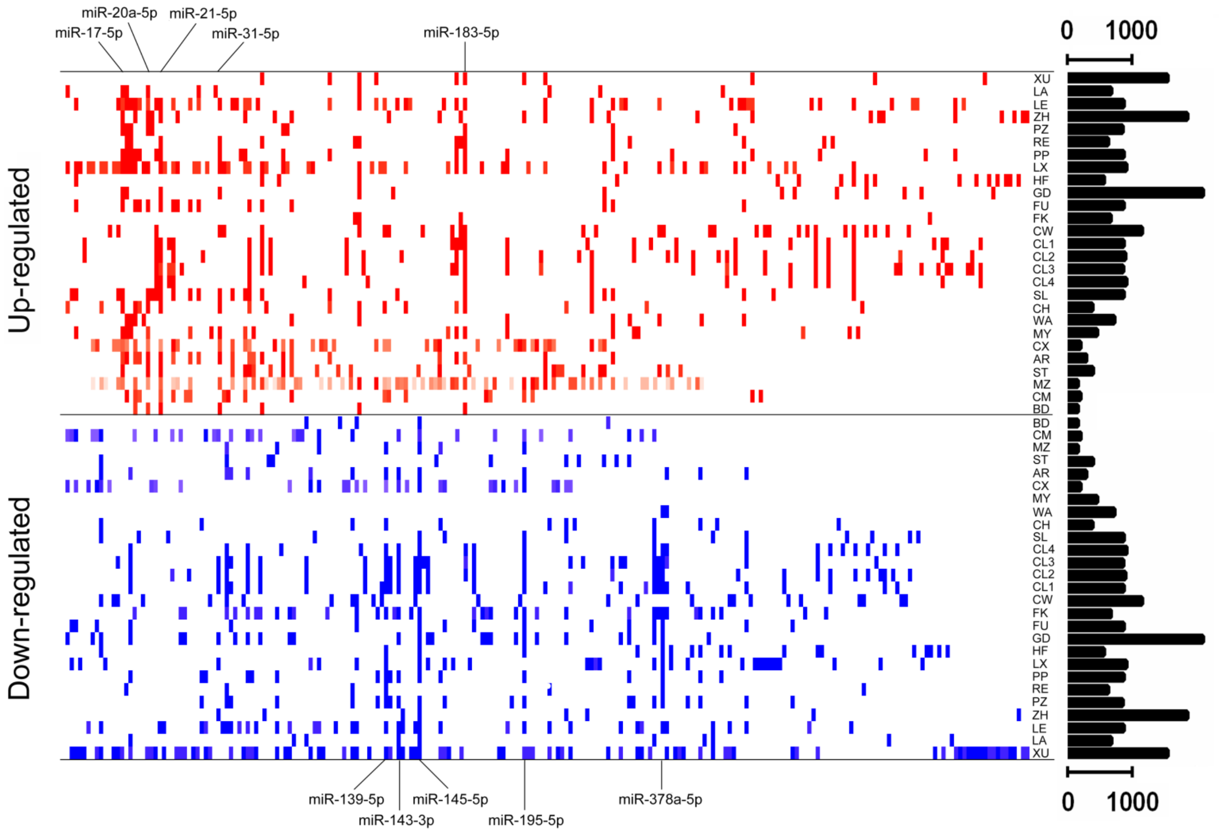

Supplementary Figure 1: Distribution of cancer-specific miRNA alterations in CRC reported by original studies. Short red and blue vertical bars represent up-regulated and down-regulated miRNAs, respectively. Studies are arranged by publication year from the center to the side. The number of miRNAs in each study is shown by a graphic portrayal on the right. The positions of CRC integrated-signature miRNAs are shown on the top and bottom.

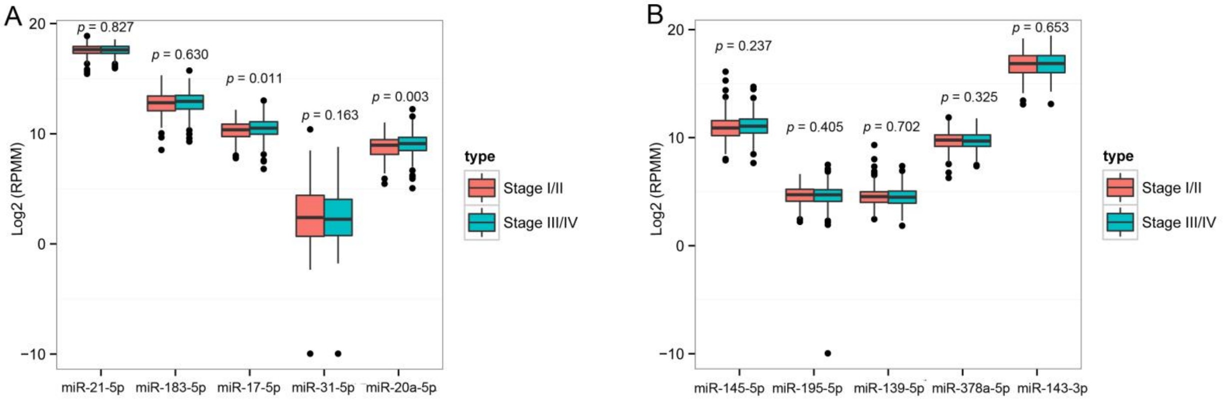

Supplementary Figure 2: MiRNAs expression in different tumor stages in the TCGA datasets. A. Upregulated miRNAs expression. B. Downregulated miRNAs expression. For boxplots, expression values of miRNAs were log2-transformed and box width was proportional to the square root of sample size in each variant.

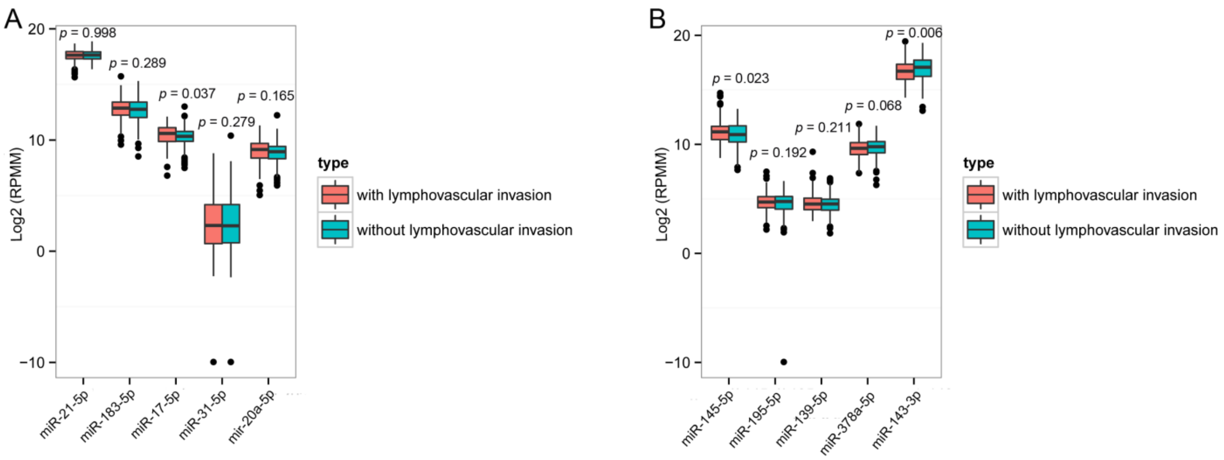

Supplementary Figure 3: MiRNAs expression with and without of lymphovascular invasion in the TCGA datasets. A. upregulated miRNAs expression. B. downregulated miRNAs expression. For boxplots, expression values of miRNAs were log2-transformed and box width was proportional to the square root of sample size in each variant.

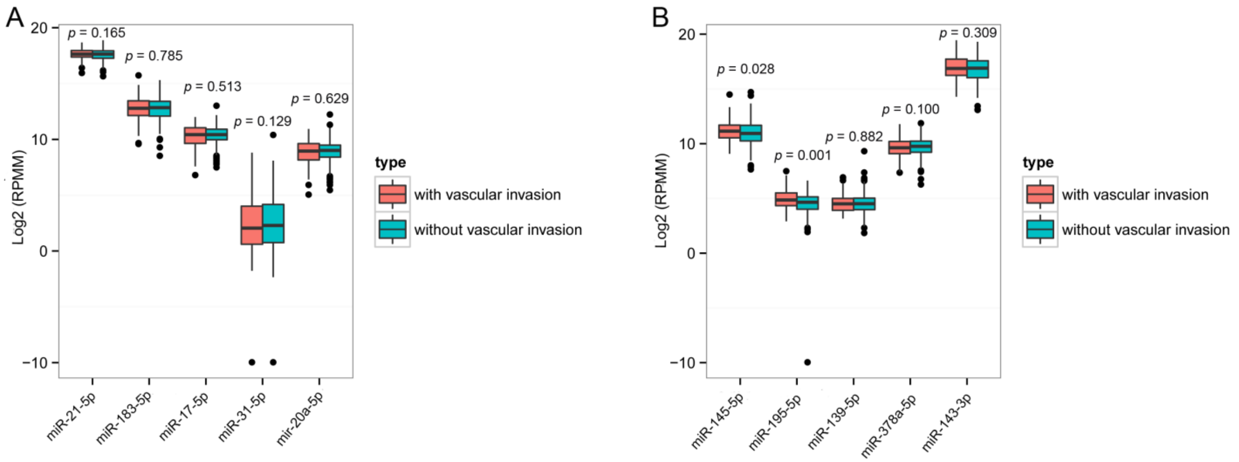

Supplementary Figure 4: MiRNAs expression in presence and absence of microscopic vascular invasion in the TCGA datasets. A. upregulated miRNAs expression. B. downregulated miRNAs expression. For boxplots, expression values of miRNAs were log2-transformed and box width was proportional to the square root of sample size in each variant.

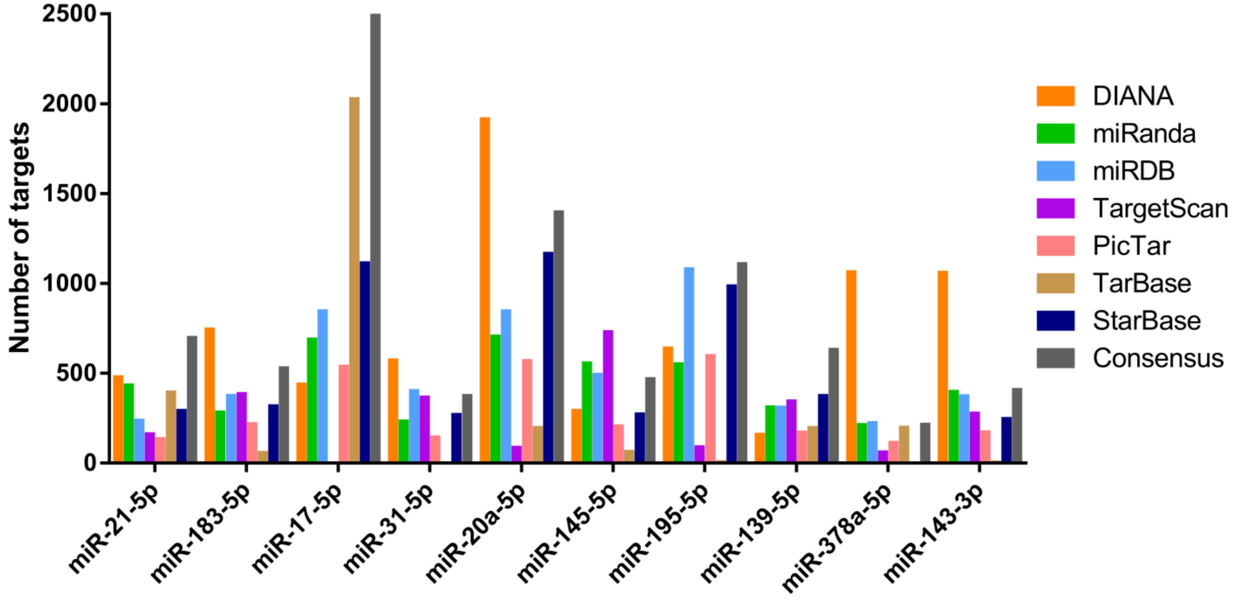

Supplementary Figure 5: The counts of predicted targets, experimentally validated targets and consensus targets of integrated-signature miRNAs.

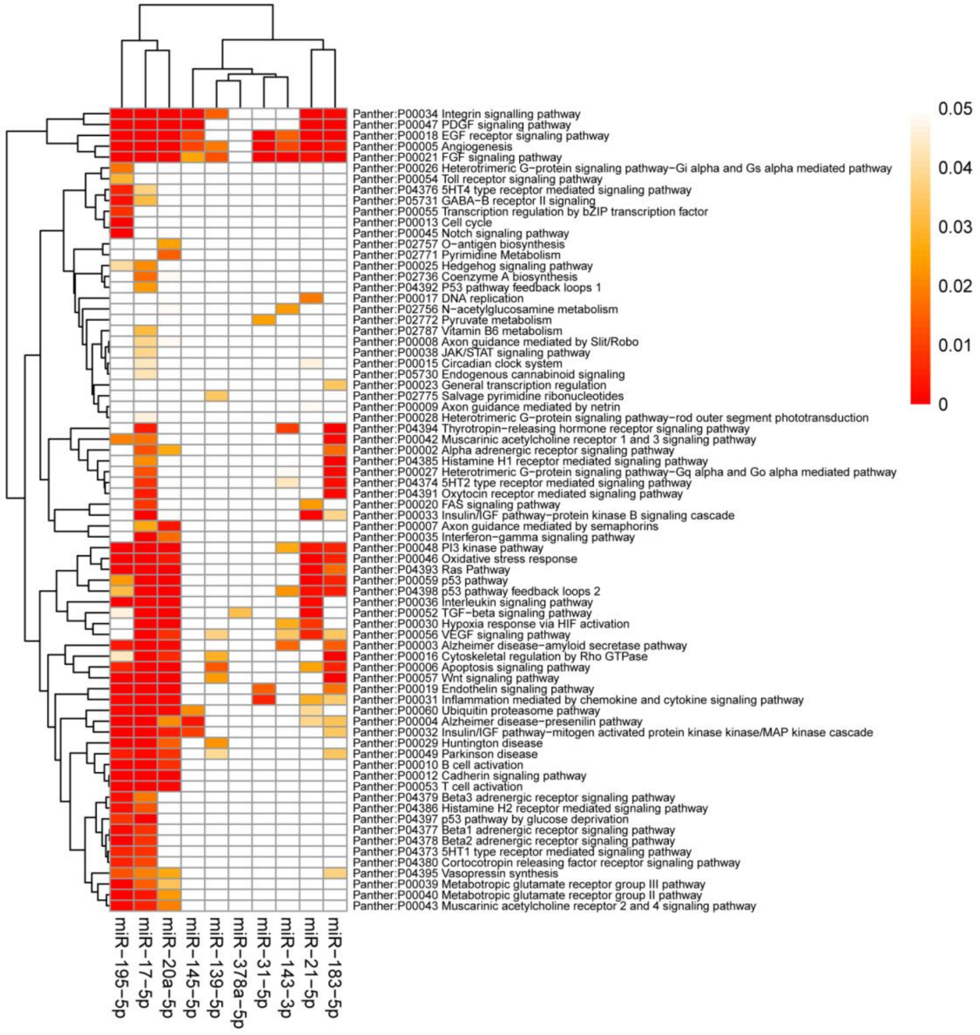

Supplementary Figure 6: The heatmap of enriched Panther pathways. Panther pathways with FDR-corrected *p*-value of each integrated-signature miRNA were constructed a heatmap showing the results of pathway enrichment analysis. The intensity of color represents the FDR-corrected *p*-value. Clustering was implemented using Pearson correlation and average linkage method.

Supplementary Table 1. Characteristics of analysed datasets

First author and publication year	Acronym	Region	Assay type	Number of miRNA probes	Tumor site	Number of samples
Xu (2014)	XU	Asia	microRNA qRT-PCR System (GeneCopoeia)	1547	Colon, Rectum	31 pairs
Liang (2014)	LA	Asia	SOLiD Sequencing (Applied Biosystems)	673	Colon, Rectum	10 pairs
Li (2014)	LE	North America	Agilent Human miRNA Microarray	866	Colon	61 pairs
Zhang (2013)	ZH	Asia	Custom Microarray	1849	Colon	40 pairs
Pizzini (2013)	PZ	Europe	Affymetrix GeneChip miRNA Array	847	Colon, Rectum	15 pairs
Reid (2012)	RE	Europe	TaqMan MicroRNA Array (Applied Biosystems)	621	Colon, Rectum	40 pairs
Piepoli (2012)	PP	Europe	Affymetrix GeneChip miRNA Array	866	Colon, Rectum	19 pairs
Li (2012)	LX	Asia	Exiqon miRCURY LNA Array	904	Rectum	6 pairs
Hamfjord (2012)	HF	Europe	Illumina Genome Analyzer II	562	Colon, Rectum	8 pairs
Gaedcke (2012)	GD	Europe	Exiqon miRCURY LNA Array	2090	Rectum	57 pairs
Fu (2012)	FU	Asia	Agilent Human miRNA Microarray	866	Colon, Rectum	8 pairs
Faltejskova (2012)	FK	Europe	TaqMan MicroRNA Array (Applied Biosystems)	667	Colon, Rectum	8 pairs
Chen (2012)	CW	Asia	Illumina miRNA Microarray	1146	Colon, Rectum	6 pairs
Callari (2012)	CL1	Europe	Agilent Human miRNA Microarray	866	Colon, Rectum	9 pairs
	CL2		Exiqon miRCURY LNA Array	891		
	CL3		Illumina miRNA Microarray	858		
	CL4		Miltenyi miRXplore Microarray	911		
Slattery (2011)	SL	North America	Agilent Human miRNA Microarray	866	Colon, Rectum	30 pairs + 40 T
Chang (2011)	CH	Europe	TaqMan MicroRNA Array (Applied Biosystems)	380	Colon, Rectum	20 pairs
Wang (2010)	WA	Europe	Agilent Human miRNA Microarray	723	Colon	3 pairs
Motoyama (2009)	MY	Asia	Agilent miRNA Array	455	Colon, Rectum	4 pairs
Chen (2009)	CX	Asia	microRNA qRT-PCR	200	Colon	6 pairs
Arndt (2009)	AR	North America	Ambion mirVana Bioarray	287	Colon, Rectum	4 pairs + 41 T
Schetter (2008)	ST	North America	Custom Microarray	389	Colon	84 pairs
Monzo (2008)	MZ	Europe	microRNA qRT-PCR	156	Colon, Rectum	22 pairs
Cummins (2006)	CM	North America	miRAGE	200	Colon, Rectum	2 pairs + 2 T
Bandres (2006)	BD	Europe	microRNA qRT-PCR	156	Colon, Rectum	12 pairs

Abbreviations: pairs, tumor tissues and paired adjacent noncancerous tissues from the same patient; T, tumor tissues.

Supplementary Table 2. All statistically significant miRNAs by integrated analysis

Up-regulated miRNA	Corrected *p*-value	Permutation *p*-value	datasets	Down-regulated miRNA	Corrected *p*-value	Permutation *p*-value	datasets
miR-135b-5p	2.81E-18	1.34E-21	13	miR-145-5p	6.13E-24	2.93E-27	21
miR-183-5p	1.57E-15	7.51E-19	17	miR-139-5p	5.04E-15	2.41E-18	16
miR-21-5p	2.03E-15	9.71E-19	19	miR-195-5p	2.25E-14	1.08E-17	17
miR-31-5p	1.94E-14	9.28E-18	14	miR-375	4.15E-12	1.98E-15	13
miR-17-5p	2.44E-10	1.17E-13	15	miR-378a-5p	2.90E-11	1.39E-14	14
miR-18a-5p	4.46E-10	2.13E-13	12	miR-143-3p	1.29E-10	6.16E-14	14
miR-96-5p	1.43E-09	6.82E-13	13	miR-133a	4.66E-09	2.23E-12	14
miR-182-5p	7.33E-09	3.51E-12	12	miR-30a-3p	5.01E-09	2.40E-12	12
miR-224-5p	1.53E-08	7.30E-12	10	miR-1-3p	1.32E-07	6.32E-11	11
miR-20a-5p	1.66E-08	7.94E-12	14	miR-30c-5p	2.12E-06	1.01E-09	11
miR-552	6.92E-08	3.31E-11	9	miR-10b-5p	3.75E-06	1.80E-09	10
miR-21-3p	5.95E-07	2.84E-10	8	miR-133b	1.73E-05	8.29E-09	8
miR-19a-3p	1.24E-05	5.93E-09	12	miR-497-5p	1.84E-05	8.81E-09	8
miR-93-5p	1.62E-05	7.75E-09	12	miR-215	0.0003	1.39E-07	7
miR-221-3p	3.37E-05	1.61E-08	9	miR-342-3p	0.0003	1.60E-07	7
miR-25-3p	5.92E-05	2.83E-08	12	miR-29c-3p	0.0009	4.21E-07	7
miR-18b-5p	7.89E-05	3.78E-08	7	miR-422a	0.0029	1.39E-06	7
miR-203a-3p	0.0002	7.80E-08	8	miR-99a-5p	0.0035	1.69E-06	6
miR-196b-5p	0.0006	2.71E-07	7	miR-30a-5p	0.0056	2.67E-06	8
miR-424-5p	0.0022	1.04E-06	6	miR-145-3p	0.0081	3.88E-06	6
miR-7-5p	0.0027	1.29E-06	6	miR-378a-5p	0.0099	4.74E-06	8
miR-183-3p	0.0027	1.31E-06	5	miR-138-5p	0.0177	8.46E-06	3
miR-106b-5p	0.0098	4.67E-06	7	miR-125b-5p	0.0186	8.91E-06	6
miR-501-5p	0.0166	7.92E-06	5	miR-147b	0.0218	1.04E-05	5
miR-92a-3p	0.0194	9.27E-06	10	miR-140-3p	0.0392	1.88E-05	6
miR-223-3p	0.0214	1.02E-05	6	miR-124-3p	0.0392	1.88E-05	5
miR-1246	0.0243	1.16E-05	3	miR-186-5p	0.0543	2.60E-05	6
miR-29b-3p	0.0303	1.45E-05	6	miR-192-5p	0.0830	3.97E-05	4
miR-106a-5p	0.0371	1.77E-05	7	miR-363-3p	0.1053	5.04E-05	4

miR-421	0.0437	2.09E-05	5	miR-628-3p	0.1082	5.18E-05	5
				miR-338-3p	0.1245	5.95E-05	5
				miR-150-5p	0.1260	6.03E-05	6
				miR-30e-5p	0.1515	7.25E-05	6
				miR-143-5p	0.1884	9.01E-05	3
				miR-9-5p	0.1932	9.25E-05	3
				miR-376c-3p	0.2074	9.92E-05	4
				miR-149-5p	0.2108	0.0001	6
				miR-451a	0.2344	0.0001	5
				miR-28-3p	0.2978	0.0001	4
				miR-190a-5p	0.3160	0.0002	2
				miR-1202	0.3196	0.0002	3
				miR-490-5p	0.3240	0.0002	2
				miR-204-5p	0.3450	0.0002	4
				miR-218-5p	0.4247	0.0002	6
				miR-638	0.4811	0.0002	6

Supplementary Table 3. Pathways highly related with CRC

Pathway	Enrichment ratio	FDR
KEGG pathway		
Kegg: 05200 Pathways in cancer	0.4537	4.61E-42
Kegg: 04010 MAPK signaling pathway	0.4427	5.02E-32
Kegg: 04144 Endocytosis	0.4767	1.80E-28
Kegg: 04120 Ubiquitin mediated proteolysis	0.5333	2.72E-26
Kegg: 04810 Regulation of actin cytoskeleton	0.4402	1.63E-25
Kegg: 04510 Focal adhesion	0.4315	6.65E-23
Kegg: 04310 Wnt signaling pathway	0.4765	2.72E-22
Kegg: 04110 Cell cycle	0.4959	1.99E-20
Kegg: 04910 Insulin signaling pathway	0.4662	5.00E-19
Kegg: 04115 p53 signaling pathway	0.6119	2.71E-18
Kegg: 04520 Adherens junction	0.5634	3.21E-16
Kegg: 03013 RNA transport	0.4069	5.91E-15
Kegg: 04350 TGF-beta signaling pathway	0.4878	1.64E-13
Kegg: 04012 ErbB signaling pathway	0.4713	3.34E-13
Kegg: 03015 mRNA surveillance pathway	0.4810	1.09E-12
Kegg: 04150 mTOR signaling pathway	0.5192	2.75E-10
Kegg: 03018 RNA degradation	0.4697	2.91E-10
Kegg: 04062 Chemokine signaling pathway	0.3172	7.01E-10
Kegg: 04540 Gap junction	0.4091	1.06E-09
Kegg: 04142 Lysosome	0.3554	3.84E-09
Kegg: 04530 Tight junction	0.3462	4.09E-09
Kegg: 04210 Apoptosis	0.3837	3.28E-08
Kegg: 04620 Toll-like receptor signaling pathway	0.3564	6.58E-08
Kegg: 04070 Phosphatidylinositol signaling system	0.3816	2.78E-07
Kegg: 04630 Jak-STAT signaling pathway	0.3007	3.14E-07
Kegg: 00562 Inositol phosphate metabolism	0.3860	6.51E-06

Panther pathway		
Panther: P00034 Integrin signalling pathway	0.4522	2.73E-20
Panther: P00005 Angiogenesis	0.4575	4.65E-20
Panther: P00047 PDGF signaling pathway	0.4844	6.65E-20
Panther: P00018 EGF receptor signaling pathway	0.5088	7.65E-20
Panther: P00021 FGF signaling pathway	0.5046	8.20E-19
Panther: P04393 Ras Pathway	0.5797	1.36E-16
Panther: P00057 Wnt signaling pathway	0.3357	1.53E-16
Panther: P00046 Oxidative stress response	0.5800	3.45E-12
Panther: P00059 p53 pathway	0.4615	4.14E-11
Panther: P00006 Apoptosis signaling pathway	0.4019	9.78E-11
Panther: P00052 TGF-beta signaling pathway	0.4176	2.86E-10
Panther: P00031 Inflammation mediated by chemokine and cytokine signaling pathway	0.3182	2.89E-10
Panther: P00036 Interleukin signaling pathway	0.4063	4.06E-10
Panther: P00048 PI3 kinase pathway	0.5098	1.42E-09
Panther: P00060 Ubiquitin proteasome pathway	0.5349	4.06E-09
Panther: P00019 Endothelin signaling pathway	0.4306	5.09E-09
Panther: P00056 VEGF signaling pathway	0.4655	7.10E-09
Panther: P00012 Cadherin signaling pathway	0.3214	6.44E-08
Panther: P00030 Hypoxia response via HIF activation	0.6154	8.48E-08
Panther: P00035 Interferon-gamma signaling pathway	0.5517	6.68E-07

Supplementary Table 4. The primers of each dysregulated miRNAs from QIAGEN

miRNAs	Product code	miRNAs	Product code
miR-21-5p	MS00009079	miR-145-5p	MS00003528
miR-183-5p	MS00031507	miR-195-5p	MS00003703
miR-17-5p	MS00029274	miR-139-5p	MS00003493
miR-31-5p	MS00003290	miR-378a-5p	MS00009646
miR-20a-5p	MS00003199	miR-143-3p	MS00003514

Supplementary Table 5: Clinical characteristics of the patients in the Tumor Cancer Genome Atlas datasets

Characteristics	No. of cases (n=580)
Age (year)	
Mean	67.9±13.2
Range	31.6-91.3
Gender	
Male	309 (53.3%)
Female	271 (46.7%)
Tumor site	
Rectum	155 (26.7%)
Colon	421 (72.6%)
Not Available	4 (0.7%)
Tumor stage	
I/II	316 (54.5%)
III/IV	250 (43.1%)
Not Available	14 (2.4%)
Lymphovascular invasion	
Yes	210 (36.2%)
No	310 (53.4%)
Not Available	60 (10.3%)
MVI	
Yes	122 (21.0%)
No	378 (65.2%)
Not Available	80 (13.8%)
Mortality	68 (11.7%)

MVI, microscopic vascular invasion
